# A novel approach to postpartum contraception: a pilot project of Pediatricians’ role during the well-baby visit

**DOI:** 10.1186/s40834-016-0018-1

**Published:** 2016-04-14

**Authors:** Rachel Caskey, Katrina Stumbras, Kristin Rankin, Amanda Osta, Sadia Haider, Arden Handler

**Affiliations:** 1 Department of Pediatrics, University of Illinois, Chicago, USA; 2 Department of Internal Medicine, University of Illinois, Chicago, USA; 3 School of Public Health, University of Illinois, Chicago, USA; 4 Department of Obstetrics and Gynecology, University of Illinois, Chicago, USA

**Keywords:** Postpartum, Contraception, Well-Baby Visit, Intervention, Pediatric Care

## Abstract

**Background:**

Postpartum women are at high risk of unintended pregnancy as many do not receive timely postpartum contraception. Utilization of routine postpartum care varies widely. Conversely, the Well-Baby Visit (WBV) for newborns is highly utilized and provides an opportunity to discuss contraception with mothers. This project aimed to test the feasibility and acceptability of having pediatric residents administer a simplified Reproductive Life Plan Tool (RLPT) with postpartum women during routine infant care.

**Methods:**

Pediatric resident physicians used the RLPT with mothers of infants 16-weeks of age or less during WBVs. The RLPT prompts physicians to ask general questions about women’s contraceptive needs and offer referral services for mothers who desire contraception services. Residents participated in a feedback session and survey to assess acceptance and perceived feasibility of using the RLPT during routine care.

**Results:**

Pediatric residents completed 50 RLPTs. Seventeen percent of eligible women accepted a referral to contraception services. During feedback sessions, pediatric residents (*n* = 18) reported comfort implementing the intervention and acceptance of the RLPT for discussing contraception. Concerns included limited time during the WBV and the potential to shift focus away from infant. On a post-intervention survey (*n* = 14), 92.9 % of physicians reported comfort in using the RLPT, and 71.4 % reported that the tool was easily understood although findings were varied regarding ease of implementing a RLPT in practice.

**Conclusions:**

Findings indicate that use of the RLPT is generally feasible during routine infant care and acceptable to pediatric resident physicians with recognition of challenges to implementation. Acceptance of a referral was low among postpartum women in this pilot study.

## Background

Postpartum women are at particularly high risk of unintended pregnancy with 10–44 % of women having an unintended pregnancy in the first year postpartum [1]. For women who do not receive contraception immediately after delivery, the six-week postpartum visit is considered an opportunity to address family planning needs. However, utilization of the postpartum visit varies widely with estimates for non-attendance ranging from 11 to 40 % [2–6]. Among low-income women in Illinois fewer than 60 % of women attend a postpartum visit between 3 and 8 weeks postpartum [7]. Further, the timing of the 6-week visit is not based on current evidence about women’s sexual activity after pregnancy and the need for timely postpartum contraception, thus placing many women at risk for a rapid repeat pregnancy [8].

In contrast to the postpartum visit, the Well-Baby Visit (WBV) is highly utilized. In 2011–2012, over 90 % of U.S. infants received visits during the first year of life [9]. The AAP recommends that healthy infants have WBVs at 3–5 days of life and at one month of age, both of which are in advance of the traditional postpartum visit, with four additional WBVs recommended before one year of age [10]. Given the earlier and more frequent use of the WBV, compared to the postpartum visit, the WBV may provide an opportunity to discuss birth spacing and postpartum contraception with mothers who may not otherwise receive this information. Research has found that women are open to pediatricians taking a role in maternal screening and referral, for example, screening for postpartum depression has been successfully implemented during well child care in the U.S. [11, 12].

Typically, mothers and pediatricians do not discuss birth spacing or maternal contraception although birth spacing directly impacts children’s health and well-being [13]. The Centers for Disease Control and Prevention (CDC) recommends use of a Reproductive Life Plan Tool (RLPT) [14] to facilitate discussion of contraceptive needs with women. To date, research using Reproductive Life Plan Tools (RLPTs) have demonstrated improved outcomes for women, however, the research has focused primarily on its use in adult primary care or family planning settings [15–20]. This pilot tested the feasibility and acceptability of using a simplified RLPT with postpartum women during routine infant care by pediatric resident physicians.

## Methods

### RLPT intervention

**Fig. 1:**
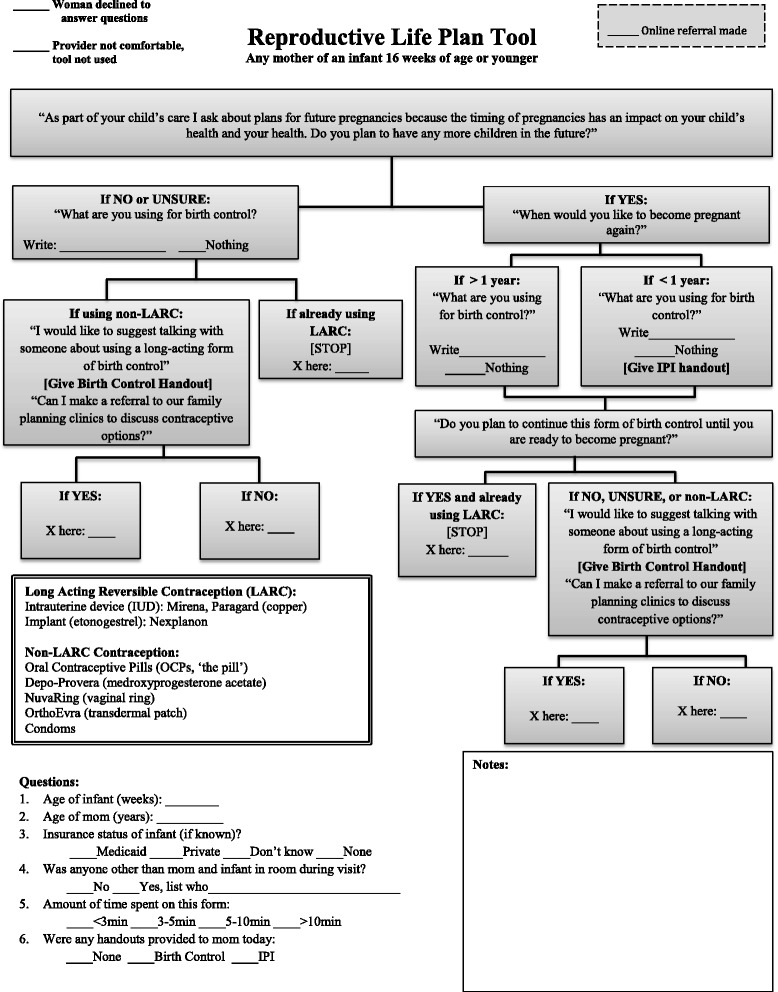
Modified reproductive life planning tool. Modified from CDC reproductive life planning tool http://www.cdc.gov/preconception/documents/rlphealthproviders.pdf

A Reproductive Life Plan Tool developed by the CDC [21] was modified (Fig. 1) to prompt pediatric residents in a large university medical center to ask about a woman’s plans for additional pregnancies and her current contraceptive needs. Any woman who reported: 1) an interest in changing her method of contraception; or, 2) no intention of ever having more children and not currently using a long-acting reversible (LARC) method of contraception, was offered a referral to family planning services. In addition, the modified RLPT included two educational prompts: a handout on recommended birth spacing provided to women who reported plans to have another child within the next 12 months; and, a handout on effective contraceptive methods provided to women who were not interested in having more children within the next year and reported not using effective contraception (in this case, defined as LARC methods), or were unsatisfied with their current methods of birth control [22, 23].

Upon completion of the pediatric visit, any woman who desired a family planning referral was directed to a computer where she could confidentially enter her contact information and request an appointment through the medical center’s appointment request website. The medical center’s Department of Obstetrics and Gynecology receives this information electronically, and typically contacts patients within three business days.

If a resident did not feel comfortable implementing the RLPT during a visit, or if a woman was not interested in discussing contraception, the residents noted this on the tool. Finally, the pediatric residents provided basic data about the woman and the visit: age of infant (weeks); age of mom (years); insurance status of infant; anyone other than mom and infant in room during visit; amount of time spent completing RLPT; and, any handouts provided. Pediatric residents did not provide contraceptive counseling or directly provide family planning services.

Implementation of the project took place in a university hospital-based general pediatric teaching clinic over a short period of time (3 weeks) to avoid multiple repeat visits that occur with young infants. Mothers of any age infant 16-weeks of age or less were eligible for the intervention. Most well baby visits in the clinic are scheduled for 20-min appointments. Twenty-five pediatric residents used the adapted RLPT during any eligible pediatric visit. Pediatric residents received an hour-long training on how to use the RLPT prior to the intervention and at least one brief refresher training during the 3 week period by research personnel who were not involved in the residency training program. Pediatric residents were instructed that their role is not to provide care to the mother, rather to use the tool to assess for those in need of care and offer a referral when needed. However, the training sessions did include a brief overview of contraceptive methods to ensure physicians were familiar with the methods. The University of Illinois at Chicago Institutional Review Board granted approval for this research.

Mixed methods were used to measure acceptability and feasibility of use of the RLPT among resident participants. All residents who participated in the intervention were invited to one of two voluntary 1-h feedback sessions. The residents gave feedback on their experience using the RLPT including ease of use, level of comfort discussing contraception during well baby care, and postpartum women’s reaction to the discussion. The groups were moderated by study personnel (AH, KS) who are not involved with the residents’ clinical training using a structured feedback group guide; the group sessions were recorded. The interviews were reviewed and themes were abstracted by two members of the research team. In addition, all residents who participated in the intervention received a confidential online survey with both open-ended and Likert-scale questions. The survey quantitatively measured individual participant’s level of comfort discussing contraception during well baby care and ease of use of the RLPT.

### Analysis

Descriptive statistics generated from the RLPTs, and the online survey results are reported using simple frequencies. Audio recordings of the feedback sessions were professionally transcribed. Detailed notes of feedback session observers (each session had one observer) were also prepared. One author (AH) integrated the main findings of the notes and the transcriptions of the feedback sessions to identify salient themes and key outcomes revealed during these sessions. Quotes that represented the themes were then extracted to support and describe findings.

## Results

Twenty-five pediatric residents (19 female, 6 male) administered 55 Reproductive Life Plan Tools representing 83 % of eligible visits; during the three-week study period, 50 tools were completed. On five occasions, either the resident (*n* =2) did not feel comfortable completing the tool or the woman (*n* = 3) declined to discuss the issues raised by the RLPT. For one of the women who declined, the resident noted the “mom had a tubal ligation, but did not want to discuss it.” No data was collected on women who declined to complete the tool. Of the two providers who felt uncomfortable using the tool, one did not wish to discuss contraception with patients’ mothers in general; the other felt the tool was inappropriate to use at a particular visit due to unrelated circumstances. The majority of the RLPTs (68.8 %) took under 3 min to complete, 29 % took 3–5 min to complete, and 2 % (*n* = 1) took 5–10 min to complete.

Among the 50 completed RLPTs, the majority of mothers were 22–35 years of age, nearly a third were one week or less postpartum, and the majority were Medicaid recipients at the time of the visit (Table 1). An additional person accompanied the mother in just over half of the visits, and the majority of these were the father of the infant (63 %). Twenty eight of all participating women reported currently using contraception and eight reported using a LARC method (5 IUD, 1 Nexplanon, and 2 unspecified). Forty-six percent of the 37 mothers who stated ‘no’ or were ‘unsure’ regarding their desire to have more children reported currently not using any contraception (Table 2).

**Table 1:** Descriptive information for postpartum women who received pilot intervention

	Total
	(*n* = 50)
Age of mother	
18–21 years	9 (18 %)
22–35 years	30 (60 %)
36+ years	6 (12 %)
Weeks postpartum	
1 week or less	16 (32 %)
2–6 weeks	19 (38 %)
7–16 weeks	15 (30 %)
Infant’s health insurance	
Medicaid (public)	31 (62 %)
Private	12 (24 %)
None	5 (10 %)
Unknown	2 (4 %)
Accompanied someone during visit	27 (54 %)
Accompanied by	*n* = 27
Infant’s father	17 (63 %)
Infant’s grandmother	5 (19 %)
Other children	2 (7 %)
Other relatives	3 (11 %)

**Table 2:** Findings from implementation of reproductive life planning tool with mothers by pediatric residents

Reproductive life planning tool data	Total
Want more children	13/50 (26 %)
Desire to be pregnant in less than 1 Year	3/13 (23 %)
Current contraceptive use	*N* = 50
Nothing	22 (44 %)
Condoms	6 (12 %)
Oral contraceptive pills	4 (8 %)
Injectable (Depo-Provera)	9 (18 %)
Long-Acting Reversible Contraception (LARC) (IUD or Implantable)	8 (16 %)
Abstinence	1 (2 %)
Provided handout on interpregnancy interval	12/50 (24 %)
Provided handout on contraceptive methods	24/50 (48 %)
Offered a referral to family planning services	36/50 (72 %)
Accepted referral to family planning services, of those eligible and offered	6/36 (17 %)

Of the 36 women eligible for a referral to family planning services, six (16.7 %) accepted the referral and all completed the online appointment request. Among the women who were eligible but declined referral (*n* = 30), nine (30 %) stated they already had an appointment scheduled or a plan to obtain contraception; 11 (37 %) reported using a non-LARC method (i.e. condoms, Depo-shot) and 10 (33 %) were not currently using contraception and did not specify a reason for not pursuing a referral. Of those who declined referral, 14 (47 %) were accompanied by someone during the visit (71 % baby’s father/mother’s significant other).

### Feedback sessions with pediatric residents

A total of 18 residents attended one of two feedback sessions (assignment to group was random). The majority of pediatric residents felt comfortable with the general idea of discussing reproductive plans with their patients’ mothers at the WBV, although most had not done so prior to participating in this pilot project. Fewer (less than a quarter) residents expressed concern regarding the discussion of contraception, and specifically LARC, with their patients’ mothers. In particular, they had concerns about not having the time or knowledge to discuss LARC (Table 3). Others expressed concern about taking attention away from the infant and emphasized that their responsibility is ultimately to the infant, and the infant’s health, above all else. Still others mentioned the limited time they had with any given patient and the difficulty of fitting ‘one more thing’ into a WBV.

**Table 3:** Key themes and quotes from feedback sessions (*n* = 2 sessions) with pediatric residents (*n* = 18)

Main themes	Key quotes
Pediatricians expressed comfort with implementing the intervention RLPT during Well-Baby Visits.	“I found [RLPT] really easy to use on those newborn visits. It was a really easy way to transition into that discussion with the parent and be, ‘Well…we want to talk about this,’ because like you guys said, if you [mother] have your visit versus baby’s visit, you’re more likely to make baby’s visit if there’s going be one.”
	“I didn’t feel uncomfortable, but it’s definitely something I never thought about doing before, I guess, in my other visits. I usually ask the mom, ‘Oh, what are you doing for your help at home?’ or, ‘Do you want to have other kids?’”
Pediatricians felt women were general comfortable discussing contraception during their child’s visit but limited in how much they opened up.	“I wonder if they [mothers] didn’t get into a discussions because it was a pediatrician and their kids’ doctor as opposed to their own doctor.”
Pediatricians were concerned with the limited time during the visit to discuss contraception.	“One thing I’m just really concerned about is obviously, I want to bring [LARC] to [the mother’s] attention, but it’s just going to open up Pandora’s Box, ‘LARC, what’s LARC?’…”.
	“While [postpartum contraception] is important and it is something that we ideally would be able to get through with everything, then again, it’s not necessarily my patient’s health. This would be put at the end of the list. If I have time to get to it, I would get to it, but with the kid in front of me, that’s my priority”.
Pediatricians had suggestions for improving the intervention including: having women complete the tool in a different setting and expanding the intervention to include women up to one year postpartum.	“I wonder if we really want to get the information out, if we would just put it in all of our Bright Futures packets’cause then they would have access to it. They would bring it home with them. I mean, again, I don’t know how many parents actually sit down and read everything in their newborn packets or Bright Futures packets, but it is another way to kind of get them information there. I think it’s easier,’cause there’s a table of contents, and sometimes I’ll circle and be like, ‘Hey, these are some great things that you should probably be thinking about,’ or whatever, and then kind of giving them much opportunity to read about it, regardless of if I’ve actually asked them specifically, ‘What is your plan?’”
	*Moderator*: “Would you recommend, then, changing the time frame for when the tool is given?”
	*Respondent*: “I might, any mom of a kid under, I don’t know, six months to a year.”

Nearly all the participants reported that women seemed comfortable discussing their contraception needs during the WBV. Residents reported that women were generally open and willing to talk about the subject and a few women went into further detail than what was required to answer the questions prompted by the RLPT (Table 3). Residents agreed that the time of day affected a woman’s comfort and willingness to discuss contraception at the WBV. Women engaged less in a discussion at clinical visits later in the day and resident’s speculated this could be due to an extended wait time for appointments later in the day. Additionally, residents noted women were less likely to feel comfortable discussing contraceptive needs if a male partner was in the room.

Residents universally agreed that mothers’ postpartum care and contraception needs rarely come up during a typical pediatric visit, but many believed that the WBV may be an opportunity to capture women who may not attend the traditional postpartum visit (Table 3). Some residents suggested expanding the intervention to include mothers of infants up to 1 year of age to include more women. Although most residents seemed to agree that postpartum care is important, some were concerned that having the pediatrician play a key role in this care may go outside of the scope of their practice.

Residents discussed the feasibility of providing a referral to family planning services, as opposed to having mothers fill out an online appointment request, as a part of their practice. Overall, the residents were open to the idea of referring mothers for clinical services but were concerned with how the referral process would be operationalized given that the mother is not their patient and may not be a patient in the University’s health care system.

Throughout the feedback group discussions, suggested improvements for the implementation of the RLPT were provided. For example, some stated that the newborn nursery visit, prior to discharge after birth, may be a natural time to facilitate a conversation about contraception with mothers. At this time, the pediatricians have easy access to obstetric providers who could provide the mother contraception or facilitate a referral for clinical services. Conversely, some residents felt that although the newborn nursery would be an easy place to conduct this intervention, this may be too early in the postpartum period as some women may not be ready to make family planning decisions.

The pediatric residents stated that the RLPT was generally straightforward, easy to incorporate into clinical care and served as a reminder to discuss postpartum contraception with mothers. However, many felt the RLPT used in this study was more wordy than necessary. Many stated that they are comfortable talking about contraception without use of a tool and suggested that it may be more helpful to have a prompt related to maternal contraception on a clinical note template. The residents suggested modifying the RLPT so that it could be self-administered by women while waiting for the pediatric visit.

Finally, the feedback session moderators presented a ‘simplified’ version of the RLPT used in the intervention and asked the residents to provide feedback. All participants agreed the simplified RLPT would be easier to use than the original intervention tool; none expressed any concern that this simplified RLPT left out information vital to the intervention. All agreed that neither tool as currently designed was appropriate for the women to use on their own and would need to be administered by the physician.

### Online survey of pediatric residents

An online survey gave the residents an anonymous opportunity to provide feedback about the intervention. Fifty-six percent of the residents (15/25) completed the survey. Respondents reported generally feeling comfortable discussing reproductive planning and contraception with their patients’ mothers. On a scale from 1 (not at all comfortable) to 10 (completely comfortable), the average ranking was 7.36, with 92.9 % of respondents reporting some level of comfort (6–10 on above scale). Residents perceived mother’s comfort discussing contraception with a pediatrician on the same scale was an average of 6.29 with 71.4 % responding some level of comfort (scores between 6 and 10 on Likert scale).

The majority of residents responded favorably with regard to the RLPT used in the intervention (Table 4). Over 70 % reported the RLPT easy to follow and understand, while three disagreed with the statements pertaining to the ease of use of the tool. Over three-quarters of respondents disagreed with the statement that the tool was too complicated. When presented with a statement that the ‘tool took too long to implement with each mother’, 42.9 % disagreed with the statement. To assess general feasibility, residents were asked if ‘it would be easy to implement the screening tool as part of their regular practice.’ The responses to this question were evenly split with 42.9 % agreeing with this statement, 42.9 % disagreeing, and 2 respondents choosing to neither agree nor disagree.

**Table 4:** Pediatric residents’ acceptability of reproductive life planning tool (*N* = 14)

	Strongly agree or Agree (%)	Neither Agree nor disagree (%)	Disagree or Strongly Disagree (%)
The tool was easy to follow	71 %	7 %	21 %
The tool was easy to understand	71 %	7 %	21 %
The tool was too complicated	14 %	7 %	79 %
The tool took too long to implement	29 %	29 %	43 %
This screening tool would be easy to implement into my regular practice	43 %	14 %	43 %

Pediatric residents were also asked if they encountered any challenges in implementing the RLPT, to which 4 pediatricians (28.6 %) responded ‘yes’. All four of these individuals cited ‘time constraints’ as the source of these challenges. When asked about what they did not like about the RLPT, four responded that it needed to be simplified or streamlined two felt it distracted from their focus on the child during the visit, and one did not feel confident in his or her ability to answer questions about contraception with mothers. Residents were also asked to report on the positive aspects of using the RLPT. One noted, “I liked the hand-outs to give mom about birth control options”. Five respondents commented on the importance of discussing contraception and attendance at the postpartum visit with the mothers of their patients and stated that the RLPT served as a good reminder to initiate this conversation.

## Discussion

This study assessed the acceptability and feasibility of pediatric resident use of a RLPT to initiate a discussion of birth spacing with postpartum women and identification of women in need of contraceptive services during the Well-Baby Visit. Findings from this pilot indicate that the RLPT is generally easy to use and acceptable to pediatric residents with few challenges in implementation. The primary concerns included the potential risk of taking time and focus away from the child, the limited amount of time available during a clinical encounter, and not feeling prepared to discuss contraception with women. However, benefits included improving timely access to contraception for postpartum women and gaining comfort discussing contraception which is a skill that could be used more broadly in practice (adolescent patients).

Women who have had a recent pregnancy are at increased risk of unintended pregnancy compared to other women of reproductive age not using contraception [24]. Pregnancies with a short interpregnancy interval (within 18 months of delivery) have been associated with increased risk of preterm birth, low birth weight, and preeclampsia [25]. Improved access to contraception during the postpartum period, particularly long-acting reversible contraception (LARC), is needed to reduce unintended pregnancies and help women achieve appropriate birth spacing. While historically the obstetric postpartum visit provided the opportunity for women to receive contraception, many women, particularly low-income women, do not attend the postpartum visit [4]. Our study successfully implemented a referral program for maternal contraceptive services within pediatric care by having pediatric residents assess postpartum women’s needs for contraception services and provide a referral, during routine infant care. We found resident physicians were able to successfully implement this assessment during care and postpartum women were overall willing to participate when asked about contraception needs during a well-baby visit. Notably, 70 % of women in our pilot project were 6-weeks or less postpartum, thus, would not yet have had a traditional postpartum visit. Perhaps one of the primary benefits of screening for postpartum contraception needs during a WBV is to capture women early in the postpartum period that may be at-risk for early unplanned pregnancy.

As expected, we found that pediatric residents acknowledge the importance of subsequent birth spacing for the health and well-being of the newborn infant. The pediatric residents felt the issue of postpartum contraception was within the purview of pediatric care as it impacts the health of the mother and family unit. Practicing pediatricians in the community who have a large clinical practice and greater administrative burden may not share this enthusiasm about adding a clinical tool to their practice. However, many pediatricians are already addressing some maternal health issues during pediatric visits. For example, providing postpartum depression screening during the newborn period has become routine practice for many pediatricians and is now reimbursed by many insurance companies and by Illinois Medicaid [26–28].

### Limitations

This pilot study has a number of limitations. Not every pediatric resident attended the 1-h training session, thus, some residents may have forgotten to complete the RLPT or found the tool took a longer amount of time to complete compared to those who had attended the training. To address this issue, residents received a brief refresher (from research personnel) at the start of many of the clinic sessions during the pilot, though the brief training session may not have been adequate for all residents. We do not know how often an individual resident used the RLPT. For example, some may have used the tool only once while others may have administered it more frequently. Additionally, the RLPT was not designed to identify women using less effective contraception. For example, women who intended to become pregnant again and were planning to continue their current method of birth control were not offered a referral to family planning services or information about LARC, regardless of whether or not they were using an effective form of contraception. Our intent in this initial pilot was to avoid the pediatrician engaging in a conversation about contraception for which they might not be comfortable, but rather to identify women most in need of family planning services. In addition, in this pilot study, delivery of family planning services was expected to be at another site on a different day, thus potentially rendering the intervention ineffective. The pilot took place in an academic medical center where a referral to family planning services is relatively convenient; this may not be the case in a community setting.

Unfortunately, we were unable to follow mothers to determine if the online referral request resulted in a family planning visit with the provision of contraception. We were also unable to incorporate feedback from women themselves about their comfort level and satisfaction with the intervention. Finally, we were unable to assess if having someone else in the exam room during the visit (e.g. infant’s father) impacted women’s answers on the RLPT or likelihood of accepting a referral.

Additionally, there are limitations related to the survey and the feedback session. We do not know if the participants who completed the anonymous online survey also participated in a feedback session. For both the feedback session and online survey it is possible that those who chose to participate were more motivated to do so, such that they were either more strongly opposed to, or supportive of, the intervention. In addition, our sample included only resident physicians at an academic teaching institution. Consequently, our findings cannot be extrapolated to how this RLPT or modifications would be adopted and implemented in a typical pediatric practice.

## Conclusions

Despite challenges in implementing the RLPT into a busy resident pediatric practice we found implementation of a Reproductive Life Planning Tool is feasible and generally acceptable to pediatric residents and postpartum women. As such, pediatricians have the potential to play an important role in facilitating receipt of needed contraception among postpartum women.

## Abbreviation

CDC: centers for disease control and prevention
RLPT: reproductive life plan tool
WBV: well-baby visit
